# Common Dietary Modifications in Preclinical Models to Study Skeletal Health

**DOI:** 10.3389/fendo.2022.932343

**Published:** 2022-07-14

**Authors:** Elizabeth Rendina-Ruedy, Brenda J. Smith

**Affiliations:** ^1^ Department of Medicine, Division of Clinical Pharmacology, Vanderbilt University Medical Center, Nashville, TN, United States; ^2^ Department of Molecular Physiology and Biophysics, Vanderbilt University, Nashville, TN, United States; ^3^ Department of Obstetrics and Gynecology, Indiana University School of Medicine, Indianapolis, IN, United States; ^4^ Indiana Center for Musculoskeletal Health, Indiana University School of Medicine, Indianapolis, IN, United States

**Keywords:** nutrition, metabolism, diets, bone, fracture

## Abstract

Bone is a highly dynamic tissue that undergoes continuous remodeling by bone resorbing osteoclasts and bone forming osteoblasts, a process regulated in large part by osteocytes. Dysregulation of these coupled catabolic and anabolic processes as in the case of menopause, type 2 diabetes mellitus, anorexia nervosa, and chronic kidney disease is known to increase fracture risk. Recent advances in the field of bone cell metabolism and bioenergetics have revealed that maintenance of the skeleton places a high energy demand on these cells involved in bone remodeling. These new insights highlight the reason that bone tissue is the beneficiary of a substantial proportion of cardiac output and post-prandial chylomicron remnants and requires a rich supply of nutrients. Studies designed for the specific purpose of investigating the impact of dietary modifications on bone homeostasis or that alter diet composition and food intake to produce the model can be found throughout the literature; however, confounding dietary factors are often overlooked in some of the preclinical models. This review will examine some of the common pre-clinical models used to study skeletal biology and its pathologies and the subsequent impact of various dietary factors on these model systems. Furthermore, the review will include how inadvertent effects of some of these dietary components can influence bone cell function and study outcomes.

## General Introduction

### Skeletal Health and Background

The human skeleton represents a major organ system that undergoes continuous breakdown and rebuilding, a process referred to as bone remodeling. In fact, it has been estimated that the adult skeleton turns over or is replaced once every ~10 years. In support of such a dynamic tissue, bone is the beneficiary of a substantial proportion of the body’s cardiac output and post-prandial chylomicron remnants, which presumably supply it with a rich source of nutrients (1). Of course, the rate of bone turnover is influenced by a multitude of factors, including age, sex, genetics, hormonal status and lifestyle factors (2, 3). It is this dynamic nature that gives bone tissue the ability to adapt to different forces, which will determine its tensile strength and elastic characteristics. These structural and material properties in turn confer the bone’s ability to resist fracture. Our appreciation of the cellular players involved in bone remodeling has become quite extensive, including the role of the bone resorbing osteoclasts, bone forming osteoblasts, and mechano-sensing osteocytes that regulated bone turnover (3). However, disruptions in these tightly processes can result in an uncoupling or imbalance in their activity that leads to skeletal pathologies.

A classic example of a clinically relevant skeletal disease is osteoporosis, frequently diagnosed based on a low bone mineral density (BMD). This can be a result of increased bone resorption and/or reduced bone formation, which ultimately results in a structural deficit of bone, leading to increased fracture risk. Osteoporosis and low bone mass (i.e., osteopenia) represent major public health problems, affecting ~54 million people in the U.S. and nearly half of all adults aged 50 and older (4, 5). Perhaps even more alarming is the recent report describing osteoporosis-related fractures as being responsible for more hospitalizations than heart attacks, strokes and breast cancer combined (6). Along with the substantial financial burden (~$19 billion/year), osteoporosis-related fractures often lead to multiple comorbidities (i.e., hypertension, infections, fluid and electrolyte imbalance), and patients frequently experience diminished quality of life due to immobility, pain, and isolation (7–9). While therapeutic options have significantly aided in the management of osteoporosis, some patients still experience undesirable, adverse side-effects, and overall patient compliance to these drug regimens is low (6, 10–12). Therefore, continued investigation into the molecular mechanisms regulating skeletal homeostasis and search for alternative prevention and treatment strategies is necessary.

Although clinical randomized control trials are the gold standard for the study of osteoporosis, they are limited by the time required to detect significant treatment effects in BMD and fracture risk, and the ability to study mechanistic alterations occurring at the tissue and cellular levels. Given these limitations of human-based research, rodent models have proved to be an invaluable tool when studying skeletal health. Rodent models, particularly mice, are an ideal system as they are relatively cheap, their genome has been sequenced, short lifespan for aged studies, and perhaps most importantly, their regulation of bone is like that of humans. In this regard, mice and rats both experience bone turnover by osteoclasts, osteoblasts, and osteocytes, albeit this remodeling unit is much faster than humans with a total remodeling unit occurring in ~1 month in mice (3). While this could be a limitation is some instances, it is also advantageous as structural changes can be observed in mice ~4-6 weeks in response to treatments. Therefore, mouse models have been widely used to study skeletal pathologies, including age-related osteoporosis, disuse osteoporosis, post-menopausal osteoporosis, secondary osteoporosis including glucocorticoid treatment, anorexia nervosa, and chronic kidney disease, as well as diabetes-related bone fragility. However, while using such models’ investigators should exercise caution as to control for, or at the very least consider, potentially confounding variables to yield reproducible, reliable data that supports major conclusions. This seems intuitive, but diet for example, is sometimes overlooked and/or not taken into full consideration. It’s true that calcium and vitamin D have long been considered key nutrients in bone health, however, even these micronutrients are sometimes underappreciated in the field. Additionally, macro- and micro-nutrient (i.e., vitamins and minerals) dietary composition along with non-nutrient dietary components such as phytochemicals are not always accounted for in the diets fed to laboratory animals. Therefore, it is the aim of this review to bring attention to commonly used preclinical, rodent models to study bone diseases and how dietary components can impact study design and/or confound results.

### History of Rodent Diet Formulations

**Figure 1:**
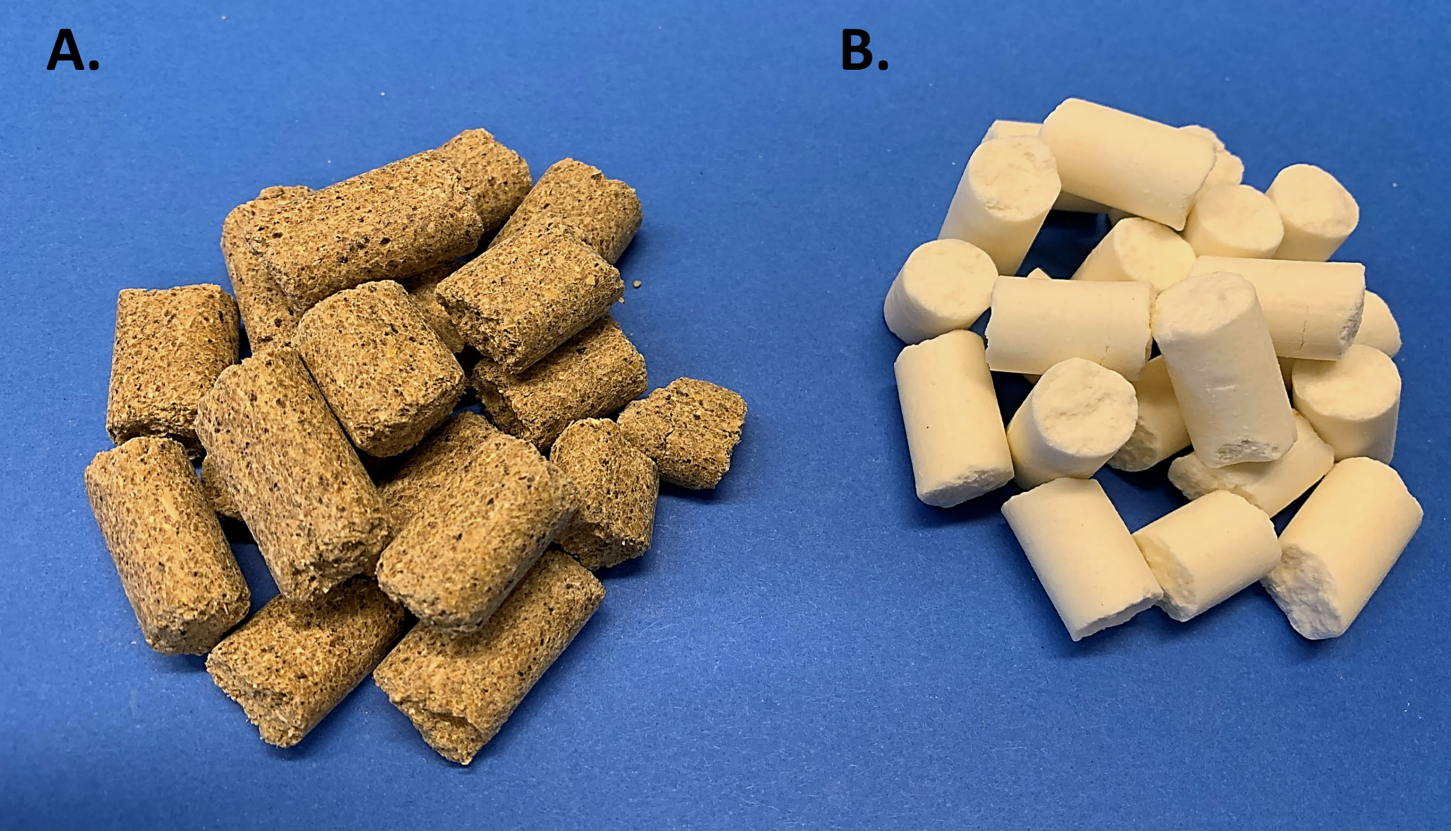
Commonly Used Rodent Diets. **(A)** Grain-based ‘chow’ diet is often closed label and varies based on environment, location, and season. **(B)** Purified diet (AIN-93M) which has documented nutritional composition and formula.

To begin, it’s important to revisit the historical perspective of rodent diets. Unbeknownst to some scientists, nutritional status of laboratory rodents was an active and heavily discussed topic in the 1970’s. In fact, in 1973 an *ad hoc* committee was formed by the American Institute of Nutrition (AIN) to identify dietary standards for laboratory rodents, which aimed to assist scientists by providing a nutritionally adequate diet that could be standardized among studies (13). This need grew out of concern from commonly used cereal or grain-based diets, referred to as “chow”, which are suspect to inherent variation (**Figure 1A**). While sufficient to sustain rodent life, chow diets are rudimentary in their nutritional composition and vary greatly depending on the manufacturer, season, and harvest location, introducing experimental variability (14). Thus, the AIN committee formulated a purified diet (**Figure 1B**), termed AIN-76, in which all components were ‘purified’ thus allowing for precise ingredient formulation and subsequent standardization. Once the AIN-76 diet started to be used, some important concerns arose, namely nephrocalcinosis or kidney calcification and insufficient blood clotting (15),. These concerns along with the fact that the diet had been based on studies with a maximum of 6 months duration and the need to periodically revisit the dietary requirements prompted an ‘AIN-76 Workshop’ to convene in 1989, which addressed these concerns, along with refining the diet to thoughtfully establish the AIN-93 diets (16, 17). After almost 3 decades of testing, the AIN-93 diets currently stand today and are available in a growth formula (AIN-93G) and adult maintenance formula (AIN-93M) (**Table 1**); however, recent discussions have highlighted the need to periodically review the diet formulation. Nonetheless, given the purified nature of this diet it establishes specified amounts and proportions of macro- and micronutrients, ultra-trace elements, along with ensuring the diet palatability and stability. Therefore, in addition to providing researchers a reproducible diet, this diet establishes a “base”, which can be easily manipulated to test scientific research questions related to nutritional aspects and model systems. In the sections to follow, rodent models commonly used for bone-related research will be highlighted, along with dietary factors that need to be considered during study design.

**Table 1:** Key nutritional components and dietary formulation of the AIN-93M diet.

Nutrient	kcal (%)	Ingredients	g/kg	Notes
***Protein***	14.7			
		Casein	140	Casein provides >85% protein. While multiple protein sources exist, casein was selected as it provides an adequate amino acid composition and is readily available. The major limitation is that casein contains a low amount of cystine, therefore, L-cystine is added to the diet. Casein also contains significant amount of phosphorous.
		L-Cystine	1.8	
***Carbohydrate***	75.9			
		Cornstarch	495.69	Starch was selected as the carbohydrate source to replace the high amounts of sucrose in the AIN76 diet, which caused many off-target effects. A diet high in starch will not pellet properly, therefore, dextrinized starch (maltodextrin) is added. A small amount of sucrose is added to provide sweetness and improve palatability.
		Maltodextrin	125	
		Sucrose	100	
***Fat***				
	9.4	Soybean Oil	40	Soybean oil provides the essential fatty acids, linoleic and linolenic acid. The amount for AIN93M diet was selected to provide an (n-6):(n-3) ratio of 7 and a polyunsaturated: saturate ratio of 4. An additional margin of safety was added for the AIN93G diet.
***Fiber***		Cellulose	50	Cellulose is wood-fiber, and while fiber is not considered a ‘nutrient’ it provides beneficial regulation of the gut microflora populations. Of the various fiber sources, iron seems to be the largest mineral contaminant.
***Minerals***		Mineral Mix*	35	Mineral mix contains essential minerals and ultra-trace elements such as calcium, potassium, phosphorous, sodium, chloride, sulfur, magnesium, iron, zinc, copper, selenium, chromium, manganese, fluoride, nickel, iodine, molybdenum, and vanadium. Mineral mix also contains powered sucrose as a dispersal medium for vitamins.
		Choline bitartrate	2.5	
***Vitamins***		Vitamin Mix*	10	This vitamin mix provides the known essential vitamins for laboratory rodents including, nicotinic acid, pantothenate, pyridoxine, thiamin, riboflavin, folic acid, biotin, vitamin B12, vitamin E, vitamin A, vitamin D3, vitamin K1. Vitamin mix also contains powered sucrose as a dispersal medium for vitamins. These vitamins are especially sensitive to light degradation.
***Anti-oxidant***		tert-Butylhydroquinone (TBHQ)	0.01	Oxidation of highly polyunsaturated oils/fats are likely and therefore, TBHQ is added to effectively prevent from oxidation. Of note, when fats are altered in diets, it is likely that additional considerations should be taken including storage temperatures and frequency of food replacement.

Each component was thoughtfully considered during the formulation process (17, 18). (*) Mineral mix and vitamin mix are specific to the AIN-93 diets.

## Preclinical Rodent Models to Study Bone and Relevant Nutritional Aspects

### Ovariectomy (OVX) Model of Post-Menopausal Osteoporosis

The lack of estrogen that occurs post-menopause is a significant risk factor for osteoporosis. In fact, one in two postmenopausal women will experience osteoporosis, and most will suffer a fracture during their lifetime (10, 19). As such, bilateral oophorectomy or ovariectomy (OVX) of rodents is a commonly used model, which mimics significant bone loss associated with the early post-menopausal period. While the use of this model is common in bone research, the nutritional nuances associated with this model are not always appreciated. For example, use of chow diet can be particularly concerning. As described above, this grain-based diet fluctuates in its components, but these diets are also formulated with soy protein/soybean meal, which contain high amounts of phytoestrogens (i.e., isoflavones). Noteworthy, many of these isoflavones such as daidzein and genistein can act by binding to estrogen receptors (ER), namely ERβ and to a lesser extent ERα, eliciting either pro- or anti- estrogenic effects (20, 21). To give context, oral administration of daidzein and/or genistein to OVX Sprague-Dawley rats has been demonstrated to reduce femoral bone loss, prevent bone loss, and even increase bone density (22, 23). Although the doses of these compounds in chow are not likely to be as high as those reported here, it is evident that these compounds, which are found in many chow diets can have a direct impact on the OVX-model unbeknownst to the researcher. Furthermore, given the variability of these compounds across chow diets, it is expected the use of these diets could yield inconsistent skeletal outcomes. Conversely, the AIN-93 diet does contain soybean oil; however, no phytoestrogens have been detected in these purified diets (24).

Another nutritional aspect to note when using the OVX model to study bone, is that OVX often results in hyperphagia or increased food intake (25–27). This is in addition to global metabolic alterations associated with the model and menopause. The increased food intake is important because many researchers have previously highlighted the complex association between adiposity and skeletal homeostasis. Therefore, alterations occurring in the skeletal could be as simple as increased weight gain and body weight. For that matter, arguably these OVX animals could demonstrate various skeletal phenotypes due to altered nutrient intake. A technique often used to control for OVX-induced increases in food intake is to match- or pair-feed OVX group to that of Sham controls. In the case of pair feeding, the amount fed to the OVX group is based on the food intake of the Sham group the prior day so that feeding is adjusted daily. In contrast match feeding, the amount of food consumed by the Sham control over a few days, (e.g., a week) would then be fed to the OVX-group the following week. Our labs and others have demonstrated that regardless of this matched diet, OVX animals gain more weight relative to Sham, but in this scenario this observation is not due simply to increased food intake and reflect alterations occurring in systemic metabolism.

### High-Fat Diet Induced Obesity (DIO) Models

One of the most striking health consequences related to the prevalence of obesity has been the staggering increase in cases of type 2 diabetes mellitus (T2DM). Over the last two decades, studies designed to determine whether T2DM influenced fracture risk based solely on the assessment of BMD revealed mixed results, with the preponderance of the evidence indicating that patients were not at increased risk (28–30). However, subsequent studies with fracture as the primary outcome variable have challenged these initial findings and the clinical evidence now indicates that patients with T2DM have an increased risk of fracture, independent of BMD (31–34). This likely results in the fracture risk of patients with T2DM being underestimated when using BMD. The apparent disconnect between BMD and fracture risk in T2DM has perplexed researchers and clinicians alike, however, a consistent finding appears to be that diabetic patients (32, 35–37) and obese animal models of T2DM (38, 39) demonstrate impaired bone turnover, particularly reduced bone formation. Therefore, these animal models provide a valuable tool for the continued investigation behind molecular mechanisms contributing to fragility fractures in T2DM.

Regarding preclinical animal models to study skeletal related outcomes associated with T2DM some debate exists relative to the “best” model system. While some investigators rely on genetically modified transgenic and congenic mouse models, others utilize nutritional interventions in the form of high fat diets. We have previously provided an in-depth review regarding this model system (40). When performing these studies some key considerations include percent fat of diet, fat source, compensatory carbohydrate source of the control diet (i.e., added fat decreases the proportion of carbohydrate and/or protein), feeding schedule, and food/calorie intake. It is important to appreciate that the term, “high fat” is relative to the standard AIN diet. Since the AIN-93M diet contains ~10% kilocalories (kcal) derived from fat (soybean oil), anything above this amount would constitute as “high” fat. Two of the more commonly used commercially available high fat diets used to induce obesity are a 45% and 60% kcal from fat diets. These high fat diets are typically formulated with less soybean oil but use lard or beef tallow to increase fat content. These lard-based high fat diets are high in saturated (myristic, palmitate, and stearic) and unsaturated (oleic and palmitoleic) fatty acids relative to the AIN-93 diets. Conversely, some labs have used the commercially available Surwit diet to induce obesity and/or glucose intolerance (41). A major difference between this diet and the previously described high fat diets is that while fat is comparable at 58% kcal, the primary fat source in the Surwit diet is hydrogenated coconut oil, which contains a high amount of medium chain, saturated fatty acids (i.e., lauric acid and myristic acid). Additionally, the major carbohydrate source in the Surwit diet is sucrose, as opposed to cornstarch in the other high fat diets. This large amount of sucrose, which is digested to yield glucose and fructose, can have a profound effect on systemic metabolism aside from the high fat content (42). As such, the carbohydrate source, along with the full dietary formula, should be considered. Another example of dietary carbohydrate modifications impacting study related outcomes is that control diets relative to the experimental selected high fat diets, must be formulated with higher carbohydrates. This can be accomplished by increasing purified carbohydrate sources such as cornstarch, sucrose, or maltodextrin. Of these ingredients, cornstarch appears to impact metabolic response the least and is generally comparable to the AIN-93 diet (18). Therefore, it’s likely that increasing the amount of sucrose to account for carbohydrates can impair glucose tolerance in the absence of weight gain (42). Therefore, if obesity is the required outcome this may not be of concern, but if obesity-related metabolic perturbations such as impaired glucose tolerance, control mice could exhibit a similar phenotype compared to experimental high fat group. It is also worth noting that high sucrose content in the diet will also produce a sweeter, more palatable food, that could impact food intake. These details again underscore the importance and care which should be taken when dietary modifications *are the* model and the need to include the details of these modifications in published reports.

### Calorie Restriction Model of Anorexia Nervosa

Anorexia nervosa is an eating disorder characterized by the severe restriction of food/nutrient intake, which results in abnormally low bodyweight and an intense fear of gaining weight. This disorder is associated with a significant reduction is BMD accounting for ~40% of patients being diagnosed with osteoporosis (92% osteopenia) and a 3-fold increase in lifetime fracture risk (43). Another striking skeletal phenotype associated with anorexia is despite the lipodystrophic response, bone marrow adipocytes increase in both their number and size (44). While the precise function of bone marrow adipocytes remains unclear, a general inverse association exists between bone marrow adipose tissue (BMAT) and BMD clinically (45). Therefore, this unique adipose depot has been of particular interest in clinical pathologies such as anorexia.

Relative to preclinical modeling, dietary manipulation in the form of calorie restriction is often used to mimic anorexia as it results in reduced BMD and expansion of BMAT. While it remains somewhat debated, a 30% reduction in total calories is often used in this model as it produces the desired outcome of reduced skeletal parameters and increases marrow adiposity (46, 47). Of note, investigators should consider formulating the diet such that a 30% reduction in calories does not result in micronutrient deficiencies. For example, we have previously used a formula which resulted in a 30% reduction in total kilocalories, but calcium and phosphate were matched to that of controls. In this regard, bone loss was evident and our ability to control these variables allowed us to determine that mineral deficiency was not the sole culprit (48). Similarly, phosphate restriction has also been shown to increase bone marrow adiposity and given the matched diet in our experiments, the same can be said for calorie restricted expansion of BMAT (49).

Other major nutritional and metabolic considerations using the calorie restriction model of anorexia nervosa involves the feast-famine feeding schedule and individual housing. Relative to the feeding schedule, animals are often food restricted during their active or dark cycle, only to be fed during the day, at which time they often consume most of their food. Therefore, the precision of the model relative to anorexia remains somewhat under debate. Additionally, animals are often individually housed to ensure each animal is consuming a known amount of food and to avoid a dominate animal from ingesting most of the food, thereby restricted others further. While controls should also be individually housed, this does introduce metabolic and behavioral disruption to the animals. Individual housing of mice has been shown to reduce growth rate while increasing energy intake and expenditure, due in part to maintain thermal neutrality without huddling of litter (50). Behavioral and endocrine alterations are also noted during individual housing and vary in degree amongst different mouse strains (e.g., BALB/c demonstrate increased anxiety-like behavior versus Swiss Webster which are considered ‘normal’); however, they should be considered when using this model (51).

### Dietary Models of Chronic Kidney Disease

The chemical composition for the mineral portion of bone, or the hydroxyapatite [Ca_10_(PO_4_)_6_(OH)_2_], inherently emphasizes the importance of mineral metabolism on skeletal health. Therefore, mineral imbalances that occur during chronic kidney diseases (CKD) significantly impact bone resulting in reduced BMD, promoting skeletal fragility and increased incidence of fracture (52). Traditional techniques for inducing renal failure have included surgical methods and currently, the most widespread methods are unilateral ureteral obstruction and 5/6 nephrectomy. Both methods lead to interstitial fibrosis by infiltration of macrophages and tubular cell death by apoptosis and necrosis, thus causes significant renal dysfunction (53). Limitations of these surgical models include the dependence on surgical skills, demand of post-operative care, high mortality rates, and reduced flexibility of dynamic urea alterations which results in the inability to study graduate disease progression (53). Additionally, dietary modification by means of using an adenine supplemented diet, commonly 0.2-0.25% adenine, has been shown to induce phenotypic CKD in rats (54). This method takes advantage of a mechanism by which adenine is oxidized *via* xanthine dehydrogenase, which yields 2,8-dihydroxyadenine. Given the low solubility of 2,8-dihydroxyadenine, stones are precipitated in the kidney tubules resulting in nephrolithiasis with extensive tubular dilation, necrosis, and fibrosis, accompanied by renal dysfunction. The adenine model has the advantage of sharing similar pathological features with human CKD (e.g., tubulointerstitial fibrosis, inflammation, glomerulosclerosis and moderate vascular calcification), with little variation between animals and develops over a relatively short period of time (54, 55). Up until recently, this model was exclusive to rats, as mice were reluctant to consume the adenine-based diets, which resulted in high morbidity and mortality due to starvation and malnutrition rather than renal failure. This limitation was recently circumvented by mixing the adenine in a chow-based diet supplemented with casein (56). In this capacity the casein effectively removed the inherent smell and taste of adenine, which resulted in mice sufficiently consuming the diet to replicate the renal dysfunction noted in rats (56).

Based on reports in the literature, some investigators supplement adenine into a purified casein-based diet, but many incorporate adenine into chow-based diets, with or without the addition of casein (56–59). The practice of incorporating nutrients into chow-based diets can still result in closed label formulations that leave researchers with little control over ingredient variability. Particularly noteworthy when incorporating the adenine into chow-based diets, these diets fluctuate in their mineral content which could significantly impact study-related outcomes. Despite this CKD model being driven by dietary alterations, its striking how few publications provide nutritional or dietary information. Additionally, some reports mention a gradual reduction in food intake with the adenine supplemented diet, but given the chow component, it remains unclear what this “reduction” accounts for in terms of actual kcal and other nutritional components. It is noteworthy that along with the CKD phenotype induced by adenine supplementation, mice also demonstrate reduced bone parameters to include expansion of BMAT. It is of particular interest whether this phenotype is directly driven by the CKD, by a relative decrease in food intake or both. Thus, monitoring of food intake is warranted. In the same vein, phosphate restriction alone has been shown to exert a similar outcome by decreasing bone formation and increasing BMAT. Therefore, when using these models, it is important to consider variables such as dietary modifications and components to fully scrutinize molecular mechanisms.

### Other Nutritional Considerations When Studying Skeletal Metabolism

In addition to the issues highlighted above, other considerations and limitations exist relative to dietary influence on preclinical rodent models while studying skeletal-related outcomes. A topic that has attracted much attention from the scientific community is that of the gut-microbiome and its influence on health and disease, to include bone homeostasis (60, 61). In fact, the gut microbiome has been shown to influence all the of the preclinical models previously discussed (62–66). Because the study of the gut microbiome often requires the use of germ-free or immune-compromised models, several dietary factors should be considered. To start, diet sterilization is required common practices include ɣ-irradiation and high-vacuum autoclaving of the diets, both of which can have profound effects on diet integrity. For example, ɣ-irradiation can result in profound losses of vitamins C, B_1_, and A, in addition to destruction of unsaturated fatty acids (67, 68). Furthermore, autoclaving rodent diet has recently been shown to increase (~3x) dietary advanced glycation end-products (AGEs) which impacts the progression of CKD (69). Therefore, the potential loss of nutrients along with potential dietary modifications should be considered when using diets that have been irradiated and/or autoclaved. Another diet issue that should be considered relative to the gut microbiome is the fiber content of diets. Grain-based chow and purified diets can vary greatly in their fiber content. Chow diets typically contain very high levels of soluble and insoluble fiber (~20% of total composition) compared to purified diets, which historically contain ~5% total fiber (14, 63). This is critical as bacteria residing in the gut produce short-chain fatty acids upon fermentation as well as other secondary metabolites which can affect bone (70). It is also worth noting, the coprophagic behavior of both rats and mice, is an important behavioral and nutritional habit used to supply essential nutrients upon a “second digestion” of fecal content. This practice results in substantially higher microbial loads in the large intestine, some 100 times higher than if coprophagia was deterred (71). Therefore, diet handling along with rodent behavior can directly impact the bioavailability of nutrients which may not have been accounted for based on original composition of the diet.

As much of this review has focused on rodent diets, another important nutritional consideration is that of food intake. This issue has been raised in conjunction with several of the models: 1) the OVX model often consumes more food relative to Sham mice; 2) the high-fat diet model of obesity, typically consume less food compared to control, but greater calories per gram; 3) the calorie restricted model relies of restriction based on ad libitum feeding group; and 4) the adenine diet of CKD often consume less due to reduced palatability/preference. However, other preclinical models used to study bone could also impact food intake including dental defects and hormonal status, and as such, care should be taken if nutritional intervention is used in the study design. Additionally, many studies within the field of bone and mineral metabolism use optical imaging of live mice fluorescent reporter mice, as in the case of multiple cancer models and with the Thermo-UCP1 promoter (72, 73). In this case, it has recently been reported that the alfalfa meal from chow diets produces a great amount of autofluorescence in the abdominal region due to the chlorophyll using the far-red and near-infrared filters (74). Therefore, its plausible that this autofluorescence can skew results, especially if time of diet ingestion is different (i.e., treatment alters food intake and/or scans done at differing times of the day).

As a final cautionary note, aside from ‘diet’, water consumed by rodent models can be a source of experimental confounders. Firstly, water can be considered a source of nutrients, namely minerals, especially in regions where the water is ‘hard’. While many animal facilities provide water that is often purified of minerals to some degree using deionization (DI) or reverse osmosis (RO), only RO can remove protozoa, viruses, and bacterium from the water. Therefore, the use of tap water is highly discouraged. Another consideration relative to the source of water is that some transgenic-mouse models use a tetracycline (Tet)/doxycycline (Dox)-inducible Cre recombination system (e.g., osteoprogenitor targeted promoters such as osterix (*Sp7*) and chondrocyte targets under the type II collagen promoter (*Col2A1*) (75, 76). While these inducible model systems provide a valuable tool to time the manipulation of gene expression, the Tet/Dox treatments are often delivered in the water and could impact bone-related study outcomes as they fluorescently label bone surface (77, 78), alter gut microbiome, and have been noted to taste bitter (79–81). This taste-aversion has even been combatted in some studies by the addition of sucrose in the water, however, this modification should be considered.

## Conclusion

**Figure 2:**
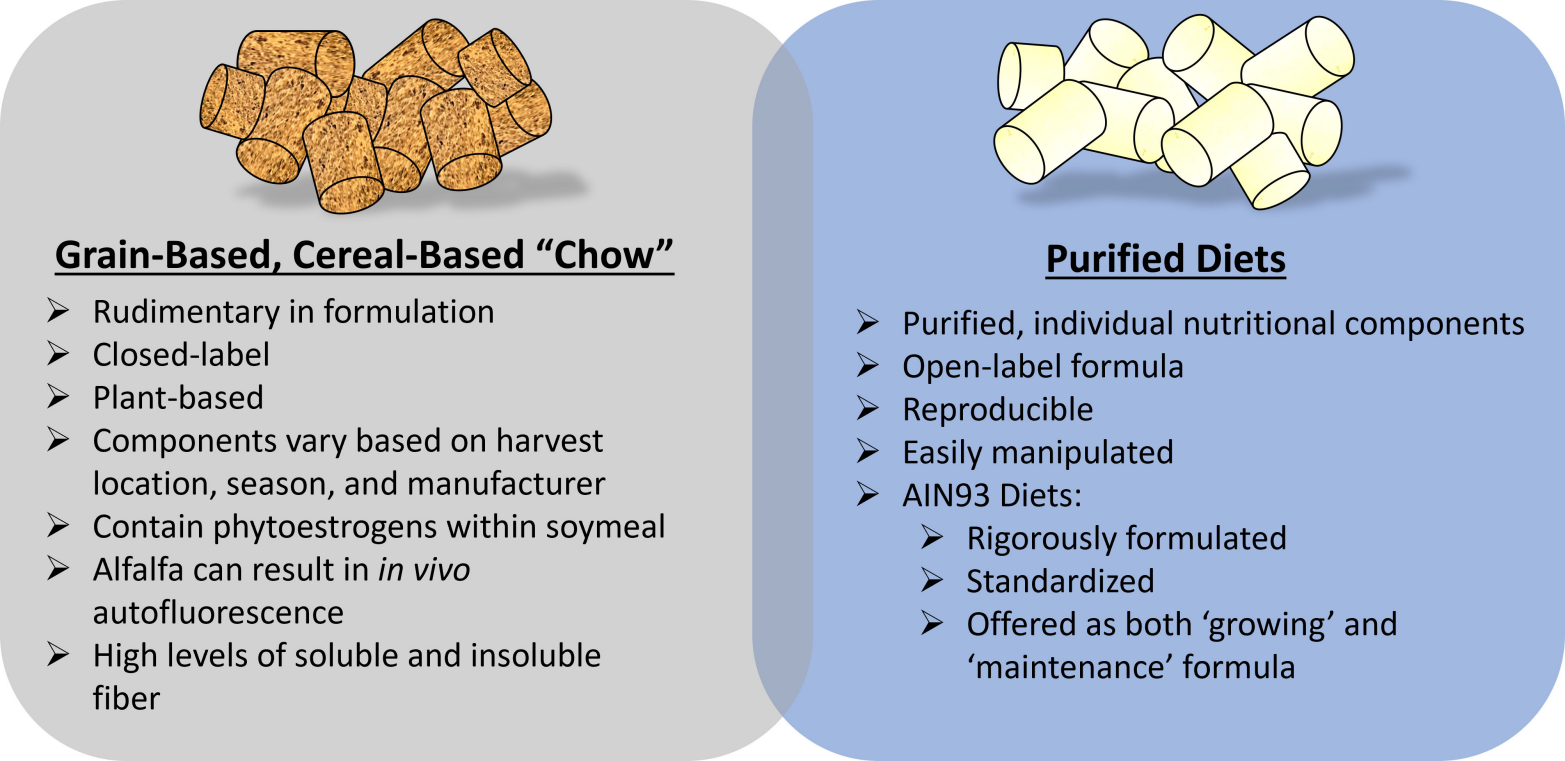
Key dietary considerations when using preclinical rodent models to study osteoporosis-related research. Fundamentally, considerations related to grain-based, chow diets versus a purified diet are important. A hybrid of these diets (chow mixed with purified ingredients) is often discouraged unless critical for study design.

When using some of the preclinical rodent models for osteoporosis research in many ways researchers assume a part of the role of ‘nutritionist’. In this capacity, researchers can ask their scientific question while controlling for potential study-confounders introduced *via* dietary sources. This review has aimed to highlight some key dietary considerations of dietary modifications when studying skeletal outcomes, **Figure 2**, however, it is not exhaustive. At a minimum, care should be taken to provide adequate dietary information when reporting results and detailing methodology, especially when the experimental model involves dietary modifications. Arguably, failure to do so is comparable to using a genetic mouse model without providing the field of ‘osteodietology’ has the exciting potential to use dietary modifications to better understand and enhance skeletal health, beyond calcium and vitamin D, key nutritional considerations must be included.

## Author Contributions

ER-R and BJS contributed to the conception of the review. ER-R wrote the first draft of the review. Both authors contributed to manuscript revision, read, and approved the submitted version.

## Funding 

This work was supported by National Institute of Health (NIH) National Institute of Arthritis and Musculoskeletal and Skin Diseases (NIAMS) Grant K01AR072123 (ERR); National Institute on Aging Grant R01AG069795 (ERR); the National Center for Complimentary and Integrative Health (NCCIH) Grant R15AT010725 (BJS) and the Office of Dietary Supplements (ODS) (BJS). The content is solely the responsibility of the authors and does not necessarily represent the official views of the NIH.

## Conflict of Interest

The authors declare that the research was conducted in the absence of any commercial or financial relationships that could be construed as a potential conflict of interest.

## Publisher’s Note

All claims expressed in this article are solely those of the authors and do not necessarily represent those of their affiliated organizations, or those of the publisher, the editors and the reviewers. Any product that may be evaluated in this article, or claim that may be made by its manufacturer, is not guaranteed or endorsed by the publisher.
